# Supplementary material for: Mapping of Genomic Vulnerabilities in the Post-Translational Ubiquitination, SUMOylation and Neddylation Machinery in Breast Cancer
Source: Cancers (Basel). 2021 Feb 17;13(4):833. doi: 10.3390/cancers13040833 (PMC7922122; doi:10.3390/cancers13040833)
Supplement: Supplementary file 1 [file cancers-13-00833-s001.pdf]

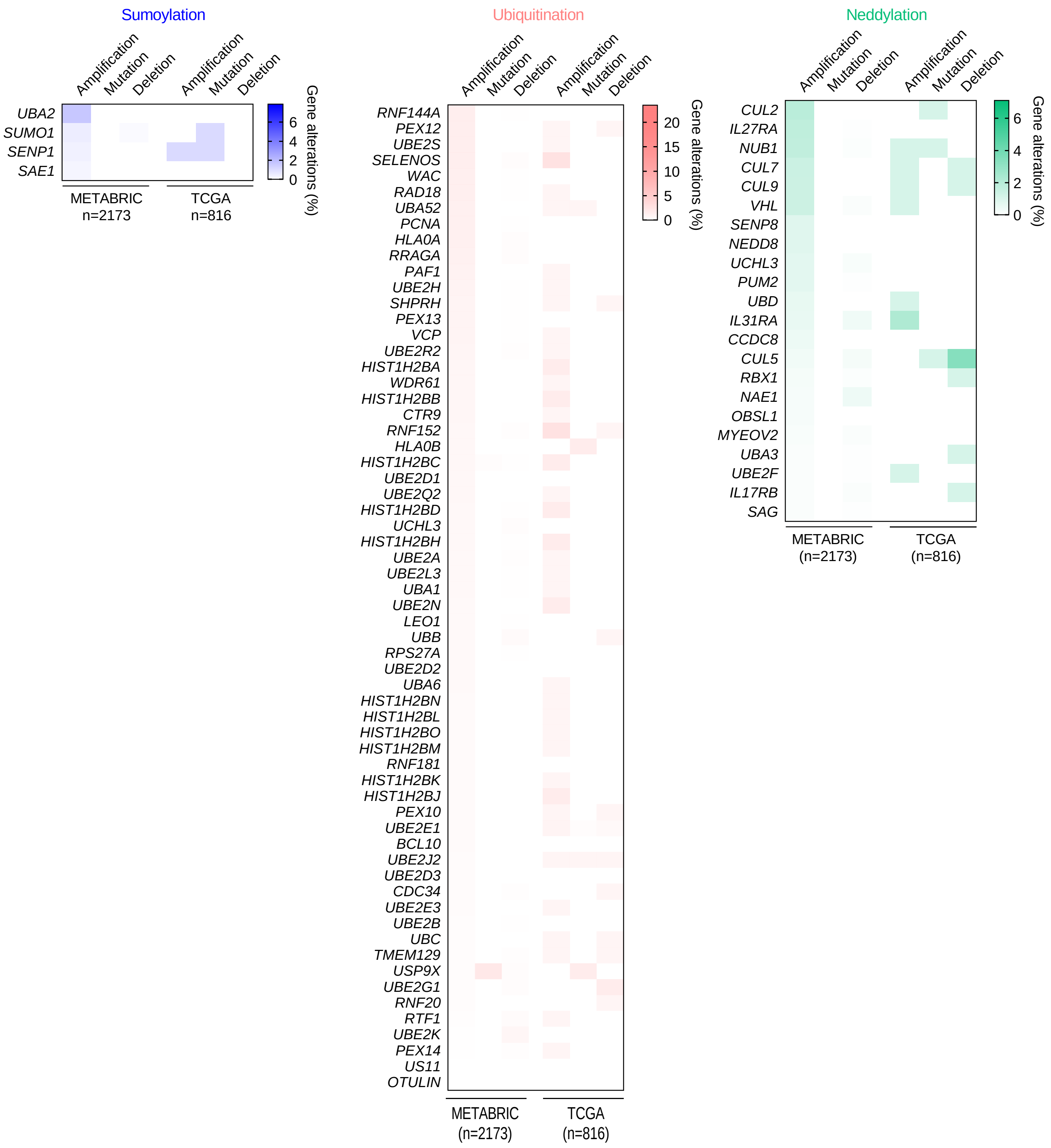

Suppl Figure 1

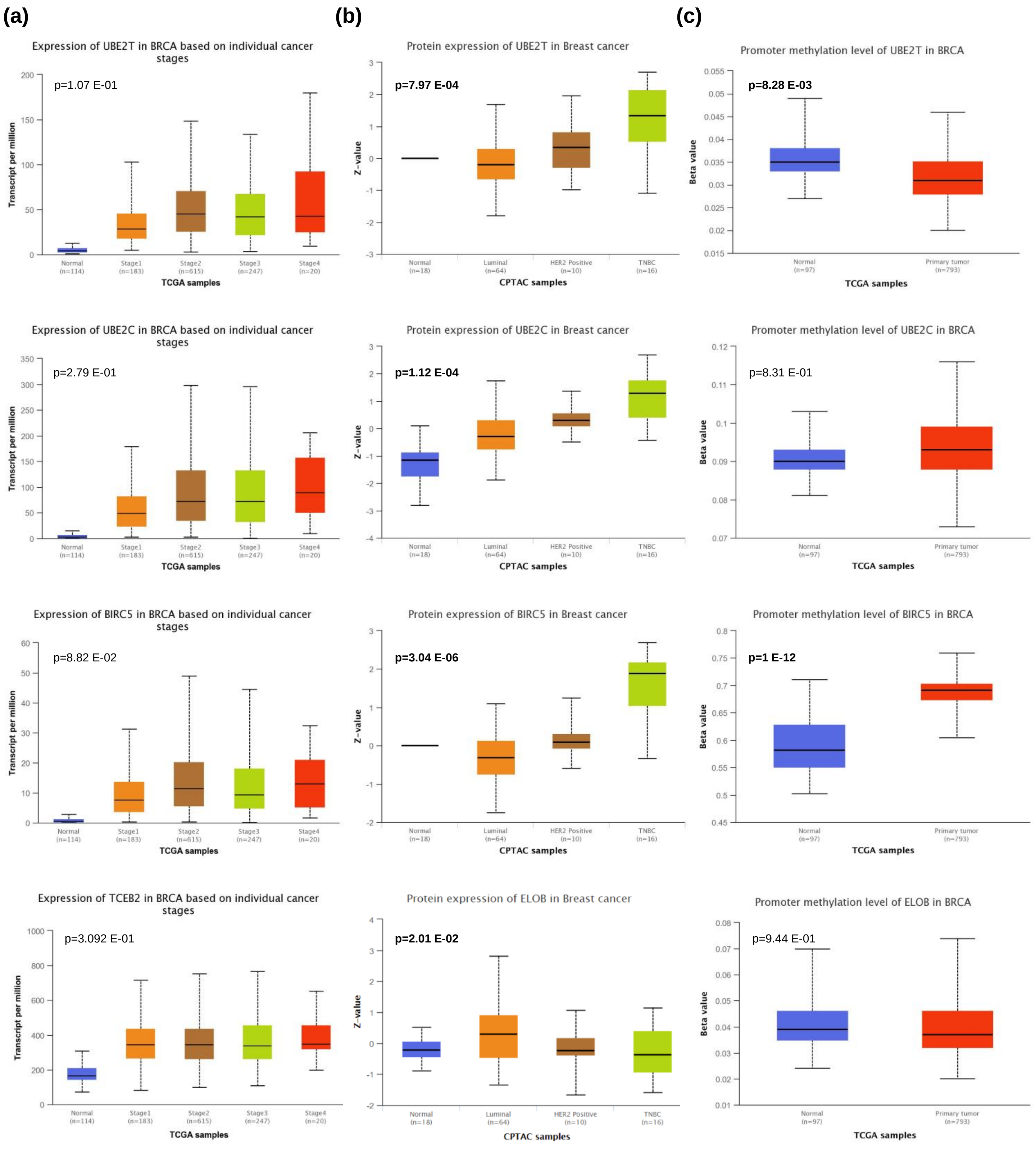

Suppl Figure 2

(a)

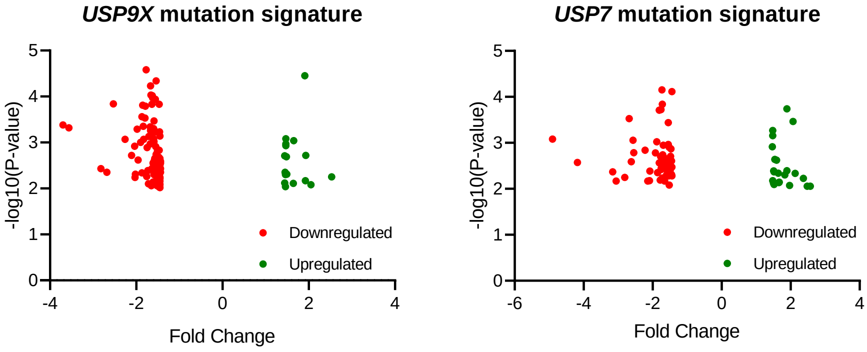

(b)

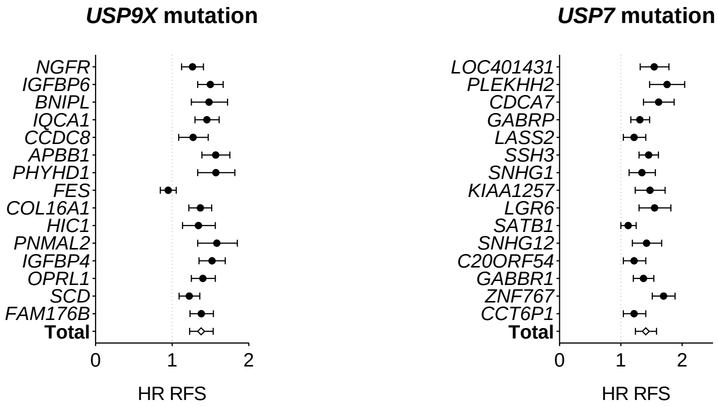

Suppl Figure 3

(a)

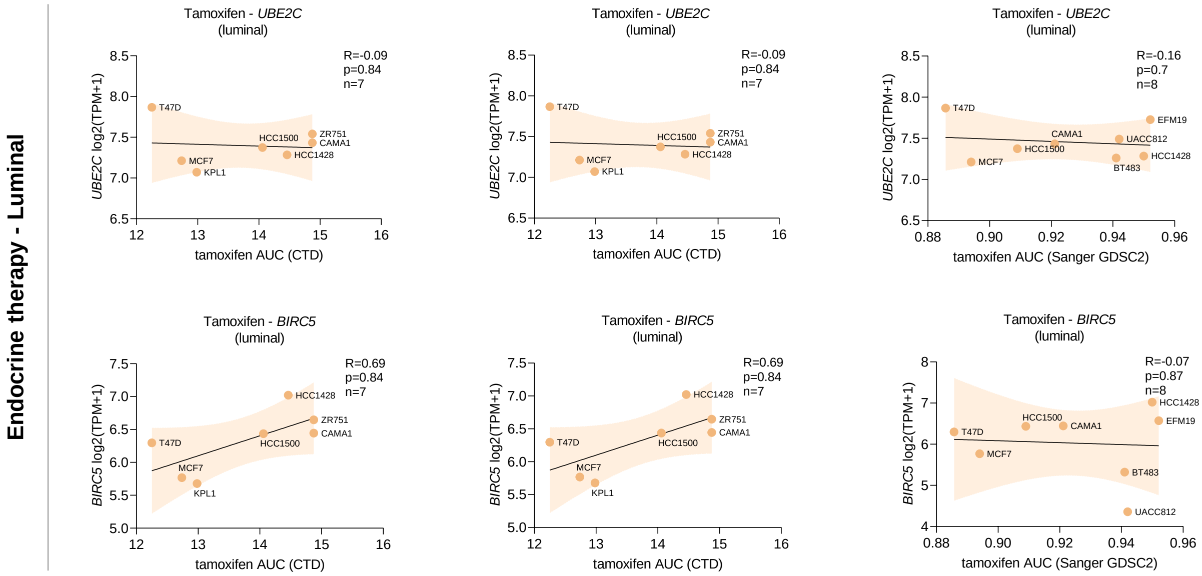

(b)

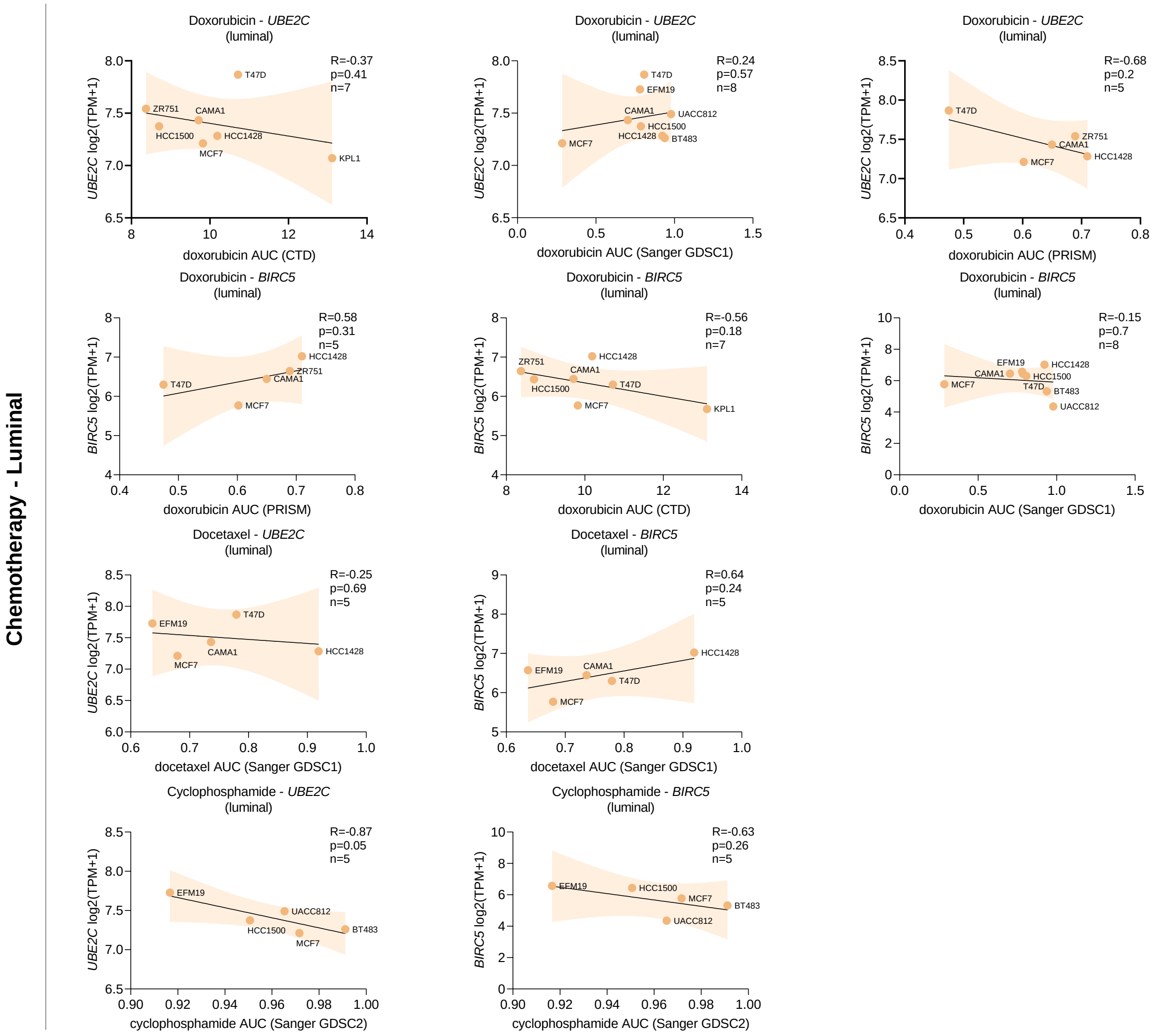

Suppl Figure 4

Chemotherapy - TNBC

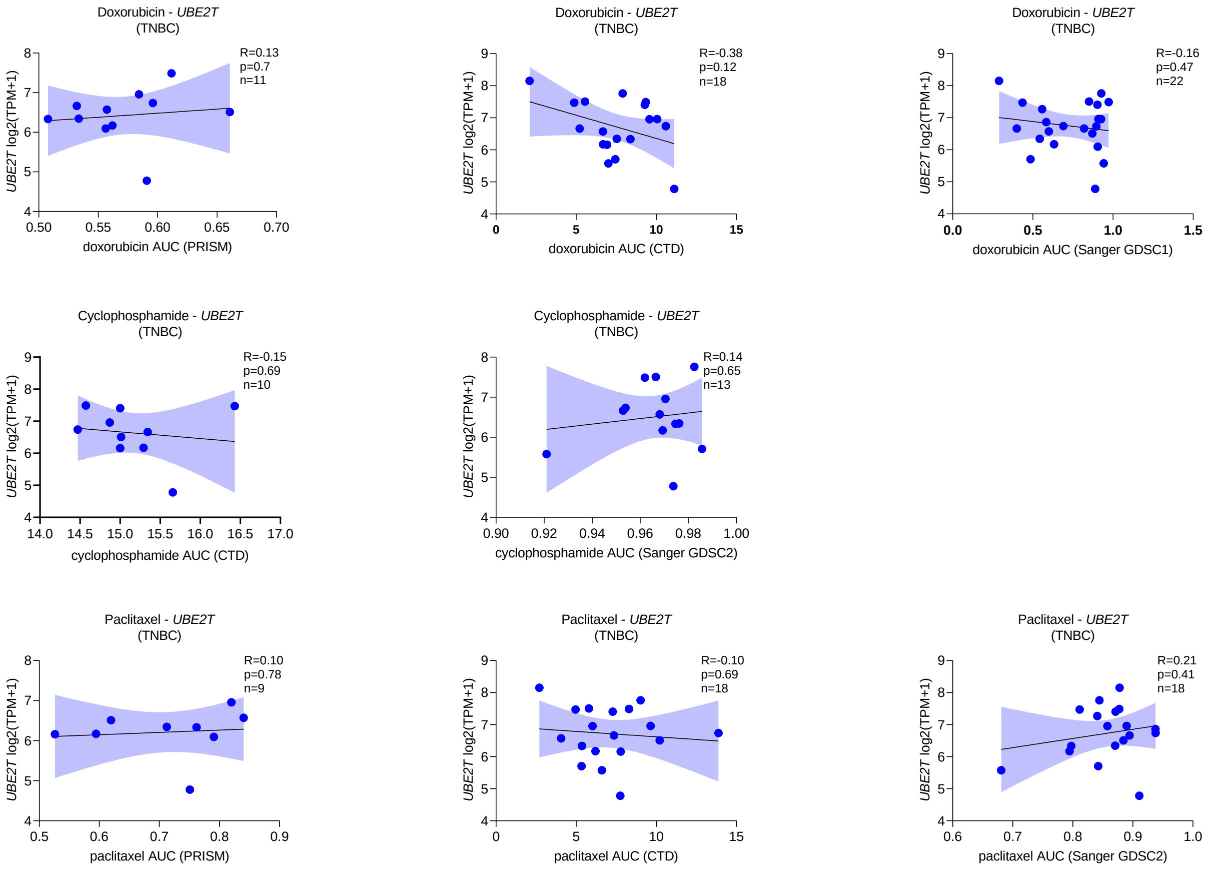

Suppl Figure 5

(a)

			SUMO2	*TCEB2*	*BIRC5*	*UBE2T*	*DERL1*	*PRKDC*	*UBE2C*
ALL	METABRIC	HR RFS	0.77 (0.61-0.98	0.82 (0.63-1.07)	1.7 (1.34-2.16)	1.7 (1.35-2.16)	1.42 (1.11-1.82)	1.4 (1.11-1.78)	1.95 (1.53-2.48)
		p	p=0.034	p=0.14	p=7.8e-06	p=7.5e-06	p=0.0057	p=0.0045	p=4.4e-08
		FDR	>50%	100%	2%	1%	>50%	>50%	1%
	TCGA	HR RFS	0.67 (0.44-1.03)	1.46 (0.95-2.26)	1.82 (1.18-2.8)	2.36 (1.4-3.99)	2.26 (1.33-3.86)	1.58 (1.03-2.44)	2.15 (1.38-3.36)
		p	p=0.069	p=0.085	p=0.0058	p=0.00087	p=0.002	p=0.036	p=0.00054
		FDR	100%	100%	>50%	20%	>50%	>50%	5%

(b)

Clinical-Pathological features	TCGA	METABRIC
**Patient (n)**	928	1976
**Mean age (Q2-Q3)**	58.1 (49.0-67.0)	61.1 (51.5-70.6)
**Nodal status**		
positive	422 (47%)	935 (47%)
negative	481 (53%)	1035 (53%)
**Tumor size (mm)**	-	26.1 (17-30)
**HER2 status**		
positive	288 (31%)	245 (12.4%)
negative	639 (69%)	1731 (87.6%)
**Molecular subtype (PAM50)**		
Basal	162 (17.5%)	328 (16.6%)
Luminal A	401 (43.2%)	718 (36.3%)
Luminal B	277 (30%)	487 (24.6%)
HER2 enriched	78 (8.5%)	238 (12%)
Normal-like	10 (1%)	199 (10.1%)
**Survival follow-up (mo)**	39.1 (14.7-53.5)	117.1 (51.4-175.5)
**Stage**		
I	167 (18%)	501 (25.3%)
II	541 (59%)	824 (41.7%)
III	209 (23%)	117 (6%)
IV	0	10 (0.5%)

(c)

		ALL	N+	N-	G3	G1
SUMO2	HR RFS	1.26 (1.13-1.41)	1.32 (1.07-1.62)	0.88 (0.72-1.08)	0.97 (0.78-1.2)	0.61 (0.36-1.04)
	p	p=3.06e-5	p=0.0089	p=0.23	p=0.76	p=0.065
	FDR	1%	>50%	100%	100%	100%
TCEB2	HR RFS	1.32 (1.18-1.48)	0.5 (0.17-1.45)	0.6 (0.31-1.16)	1.35 (1.05-1.74)	0.63 (0.37-1.06)
	p	p=7.4e-7	p=0.19	p=0.13	p=0.02	p=0.08
	FDR	1%	100%	100%	>50%	100%
BIRC5	HR RFS	1.55 (1.36-1.76)	1.64 (1.34-2)	1.57 (1.3-1.89)	0.78 (0.62-0.98)	2.22 (1.31-3.76)
	p	p=9.4e-12	p=8.9e-07	p=2.9e-06	p=0.036	p=0.0022
	FDR	1%	1%	1%	>50%	50%
UBE2T	HR RFS	2.21 (1.89-2.59)	1.82 (1.38-2.39)	1.94 (1.28-2.92)	0.68 (0.47-0.98)	2.72 (0.61-12.15)
	p	p<1e-16	p=1.6e-05	p=0.0013	p=0.04	p=0.17
	FDR	1%	1%	20%	>50%	100%
DERL1	HR RFS	1.31 (1.12-1.54)	1.55 (1.2-1.99)	1.47 (1-2.15)	1.43 (1.03-1.99)	3.97 (0.52-30.32)
	p	p=0.00072	P=0.00069	p=0.05	p=0.032	p=0.15
	FDR	10%	20%	>50%	>50%	100%
PRKDC	HR RFS	1.38 (1.23-1.56)	0.79 (0.65-0.96)	1.34 (1.12-1.61)	1.19 (0.94-1.51)	0.53 (0.31-0.89)
	p	p=4.9e-8	p=0.017	p=0.0017	p=0.15	p=0.015
	FDR	1%	>50%	50%	100%	>50%
UBE2C	HR RFS	1.82 (1.63-2.04)	2.25 (1.75-2.9)	1.91 (1.6-2.27)	0.91 (0.73-1.13)	2.51 (1.49-4.21)
	p	p<1e-16	p=7.4e-11	p=2.8e-13	p=0.39	p=0.00032
	FDR	1%	1%	1%	100%	5%

Suppl Table 1

		LUM A			LUM B		BASAL
		UBE2T	*UBE2C*	*BIRC5*	*UBE2T*	*UBE2C*	*UBE2T*
METABRIC	HR RFS	1.5 (1-2.27)	1.66 (1.1-2.49)	1.75 (1.12-2.74)	1.73 (1.18-2.56)	2.08 (1.42-3.04)	1.56 (0.89-2.73)
	p	p=0.05	p=0.014	p=0.013	p=0.0049	p=0.00013	p=0.12
	FDR	100%	>50%	>50%	>50%	10%	100%
TCGA	HR RFS	3.01 (1.46-6.23)	3.45 (1.64-7.28)	2.6 (1.25-5.4)	5.86 (0.78-43.75)	2.99 (0.88-10.16)	0.42 (0.14-1.24)
	p	p=0.0018	p=0.00053	p=0.0081	p=0.051	p=0.065	p=0.11
	FDR	50%	>10%	>50%	>100%	100%	100%

Suppl Table 2

Gene	Variation	Consequence	SIFT	PolyPhen	MutationTaster	FATHMM-MKL	CLINVAR
USP7	ENST00000344836.4:c.3011G>A	missense	deleterious	probably damaging	disease causing	damaging	NR
	ENST00000344836.4:c.2941G>C	missense	tolerated	benign	disease causing	damaging	NR
	ENST00000344836.4:c.1861C>T	stop	NR	NR	disease causing	damaging	NR
	ENST00000344836.4:c.1223G>A	missense	deleterious	probably damaging	disease causing	damaging	NR
	ENST00000344836.4:c.556G>A	missense	tolerated	benign	disease causing	damaging	NR
	ENST00000344836.4:c.230G>A	missense	tolerated	benign	disease causing	damaging	NR
USP9X	ENST00000324545.8:c.273_274del	frameshift	NR. Expected deleterious				NR
	ENST00000324545.8:c.283G>C	missense	deleterious	probably damaging	disease causing	damaging	NR
	ENST00000324545.8:c.1969C>T	missense	deleterious	probably damaging	disease causing	damaging	NR
	ENST00000324545.8:c.2084_2090del	frameshift	NR. Expected deleterious				NR
	ENST00000324545.8:c.2225G>A	missense	deleterious	probably damaging	disease causing	damaging	NR
	ENST00000324545.8:c.2248C>T	stop	NR	NR	disease causing	damaging	NR
	ENST00000324545.8:c.3479C>G	missense	tolerated	benign	disease causing	damaging	NR
	ENST00000324545.8:c.3805G>C	missense	tolerated	benign	disease causing	damaging	NR
	ENST00000324545.8:c.3876G>A	missense	deleterious	benign	disease causing	damaging	NR
	ENST00000324545.8:c.3894G>C	missense	deleterious	probably damaging	disease causing	damaging	NR
	ENST00000324545.8:c.4104_4105del	frameshift	NR. Expected deleterious				NR
	ENST00000324545.8:c.5331+1G>A	splice donor	NR. Unknown				NR
	ENST00000324545.8:c.5800A>C	missense	tolerated	benign	disease causing	damaging	NR
	ENST00000324545.8:c.6586C>G	missense	deleterious	probably damaging	disease causing	damaging	NR

Suppl Table 3

		Amplification			Mutation	
Cell line	Int Sub	*UBE2T*	*UBE2C*	*BIRC5*	*USP9X*	*USP7*
HCC1428	LA					
CAL-120	TN					
HCC1500	Incons					
MDA-MB-436	TN					
HCC1187	TN					
MDA-MB-157	TN					
HCC38	TN					
MDA-MB-231	TN					
CAMA-1	LA					
BT-549	TN					
MRK-nu-1	none					
HCC1419	Incons					
MDA-MB-468	TN					
HCC1937	TN					
MDA-MB-415	LA					
BT-483	LA					
DU-4475	TN					
HCC1395	TN					
EVSA-T	Incons					
HCC2218	HER2					
CAL-51	TN					
HCC70	TN					
CAL-85-1	TN					
JIMT-1	HER2					
MFM-223	TN					
ZR-75-30	LB					
HCC2157	TN					
HCC1143	TN					
HDQ-P1	TN					
COLO-824	none					
UACC-812	LB					
Hs-578-T	TN					
MCF7	LA					
EFM-192A	LB					
AU565	HER2					
HCC1569	HER2					
MDA-MB-175-	LA					
BT-474	LB					
T47D	LA					
HCC1599	TN					
HCC1954	HER2					
EFM-19	LA					
BT-20	TN					
CAL-148	TN					
UACC-893	HER2					
MDA-MB-453	TN					
OCUB-M	none					
MDA-MB-330	LB					
MDA-MB-361	LB					

Suppl Table 4
